# COL3A1 rs1800255 polymorphism is associated with pelvic organ prolapse susceptibility in Caucasian individuals: Evidence from a meta-analysis

**DOI:** 10.1371/journal.pone.0250943

**Published:** 2021-04-30

**Authors:** Ke Niu, Xu Chen, Yongxian Lu

**Affiliations:** 1 Department of Obstetrics and Gynecology, Seventh Medical Center, Chinese PLA General Hospital, Beijing, People’s Republic of China; 2 Center of Clinical Laboratory, First Affiliated Hospital of Soochow University, Suzhou, People’s Republic of China; Shanghai Jiao Tong University, CHINA

## Abstract

**Background:**

The collagen 3 alpha 1 (COL3A1) rs1800255 polymorphism has been reported to be associated with women pelvic organ prolapse (POP) susceptibility, but the results of these previous studies have been contradictory. The objective of current study is to explore whether COL3A1 rs1800255 polymorphism confers risk to POP.

**Methods:**

Relevant literatures were searched by searching databases including Pubmed, Embase, Google academic, the Cochrane library, China National Knowledge Infrastructure (CNKI). Search time is from database foundation to March 2021.

**Results:**

A total of seven literatures were enrolled in the present meta-analysis, including 1642 participants. Overall, no significant association was found by any genetic models. In subgroup analysis based on ethnicity, significant associations were demonstrated in Caucasians by allele contrast (A vs. G: OR = 1.34, 95%CI = 1.03–1.74,), homozygote comparison (AA vs. GG: OR = 3.25, 95%CI = 1.39–7.59), and recessive genetic model (AA vs. GG/GA: OR = 3.22, 95%CI = 1.40–7.42).

**Conclusions:**

The present meta-analysis suggests that the COL3A1 is a candidate gene for POP susceptibility. Caucasian individuals with A allele and AA genotype have a higher risk of POP. The COL3A1 rs1800255 polymorphism may be risk factor for POP in Caucasian population.

## Introduction

Pelvic organ prolapse (POP) is a phenomenon that pelvic organs such as uterus, vagina, bowel and bladder leave out of their normal anatomical location, resulting in a serious of functional disorders [1]. It is a very frequent disease in postmenopausal women, especially in senile females [2]. In recent years, the incidence of United States has increased to 39.8% in postmenopausal women [3]. However, only approximately 3% women have symptoms of vaginal bulging in some developed countries [4]. This wide discrepancy occurs as the majority of POP is symptomless. At present, the treatment of POP is mainly through pelvic floor muscle function exercise and surgery, but the effect is not very ideal. If the occurrence and pathology process of POP can be clearly defined, the fundamental treatment will bring great benefits to the patients.

Many people attributed it to some risk factors including aging, obesity, vaginal delivery, hypoestrogenism, preoperative hysterectomy, nutrition, an many other environmental factors [5–9]; however, these factors are not acting as a necessary role for POP progression. Approximate 50% POP patients do not embrace vaginal delivery or hypoestrogenism, and not everyone who is old or has preoperative hysterectomy will acquire POP. In addition, this disease can occur in young, non-pregnant women, and even virgins. People with a family history of POP might be at increased risk [10], suggesting the significance of predisposing hereditary factors. Many studies found that the majority of genetic variations relative with POP were located in genes encoding elements of connective tissue fibers or proteins associated with their composition and degradation [11, 12]. Therefore, genetic changes or variations in tissue structure, abnormal expression of the corresponding proteins might alter the pelvic organs supportive structures, leading to prolapse.

As is well known, the occurrence of POP is closely related to the integrity of pelvic floor support structure. The supportive structure of the pelvic floor depends largely on the extracellular matrix collagen. Of all the collagen, type I and type III are foremost structural fibers, which are closely associated with sidewalls of uterus, vagina and pelvic. The increased level of type III collagen is found in women who were suffering from POP [13]. During the process of delivery or other processes which lead to increasing intra-abdominal pressure, type III collagen is the first to play a role in the healing process [14]. To date, type III collagen has been the most widely investigated collagen type in pelvic connective structures, which plays an important role in the formation of vaginal wall.

Type III collagen, encoded by COL3A1 gene, consists of three identical α1 (III) chains [15]. The genetic polymorphisms of COL3A1 gene lead to amino acid change of the α1 (III) chain. Consequently, the mechanical functions of type III collagen may be influenced and pelvic floor supportive structures may be interfered. The collagen 3 alpha 1(COL3A1) gene which encodes the α1 chains of type III is located on chromosome 2q24.3-q31. Single nucleotide polymorphism is a helpful tool for mapping genes for variety of diseases. Of all the polymorphisms, collagen type III alpha 1 (COL3A1) rs1800255 polymorphism has been mostly investigated [16–22]. However, many studies have shown inconsistent or conflicting findings [16–22]. Additionally, the association between COL3A1 rs1800255 polymorphism and POP risk has not been investigated in large genome-wide association studies (GWASs). Therefore, we conducted a meta-analysis for the first time to estimate whether COL3A1 rs1800255 polymorphism confers risk to POP.

## Materials and methods

### Data collection

Two independent authors systematically and comprehensively searched Pubmed (https://www.ncbi.nlm.nih.gov/pubmed/), EMBASE (https://www.embase.com/), the Cochrane library(https://www.cochranelibrary.com/), Google academic (https://scholar.google.com/), and Chinese national knowledge internet (https://www.cnki.net/). Search time is from database foundation to March 2021. The keywords applied in the search process were as follows: “polymorphism” or “polymorphisms” or “genome wide association study” or “genome-wide association studies” or “GWAS” or “rs1800255”, “Pelvic organ prolapse” or “POP”. The literature language was limited to English language and Chinese language. Additionally, in order to avoid the omission of relevant literatures, we searched the references as much as possible.

### Inclusion and exclusion criteria

The inclusion criteria must meet a series of conditions: (a) a case-control study; (b) making an assessment of association between COL3A1 rs1800255 polymorphism and POP risk; (c) offering sufficient data or information to count OR and 95%CI. The literatures are excluded when they fit one or more of these factors of exclusion criteria: (a) case reports, reviews, meta-analysis, letters, and editorials; (b) not offering sufficient data for the present meta-analysis. (c) its experiment objective was pig, rat or other animals.

### Data extraction and methodological quality assessment

All the necessary information was independently searched, reviewed and assessed by two authors (Ke Niu; Xu Chen;). Then this contradictory data or information was reassessed by the corresponding author (Yongxian Lu). The extracted data consisted of author name, publication year, genotyping methods, sample size, ethnicity, matching criteria, source of control, HWE conformity; Newcastle-Ottawa Scale (NOS) score [23]. If the similar opinion could not reach in the course of data extraction, suggestion was offered by another experienced researcher (Yongxian Lu) to determine the correct selection. The similar method was applied equally to evaluation of literature quality. In the present meta-analysis, we applied the risk assessment criteria of Newcastle-Ottawa Scale (NOS) bias to evaluate the quality of each literature. It should be noted that the ethics approval of our study was waived by Ethics Committee of Fourth Medical Center, Chinese PLA General Hospital as no human or animal was directly enrolled in our study and meta-analysis is the statistical analysis of large collection of analysis results from individual studies for the purpose of integrating the findings.

### Statistical analysis

The association power was assessed through the corresponding indexes including OR and 95%CI. And both the Q-statistic and I^2^ statistics would be applied [24]. Four genetic models were applied in the present meta-analysis including allele contrast (A versus. G), homozygote comparison (AA vs. GG), recessive genetic model (AA versus. GA/GG) and dominate genetic model (AA/GA versus. GG). The model of fixed-effects and random-effects would be put into use on the basis of heterogeneity degree [25, 26]. I^2^ <50% was considered to low heterogeneity, 50 ≤I^2^ <75% was considered to moderate heterogeneity and I^2^ ≥75was considered to significant heterogeneity. Furthermore, the Galbraith plot was used to spot the outliers to find out the potential heterogeneity as much as possible. Sensitive analysis was applied to detect the influential studies which might contribute obvious bias to final results. The funnel plot and Egger’s test were put into use to recognize the existence of publication bias [27]. The Stata 12.0 would be responsible for the whole statistics. The present meta-analysis was performed and reported according to the Preferred Reporting Items for Systematic Reviews and Meta-Analyses (PRISMA) 2009 checklist (S1 Table) [28].

## Results

### General information

The flow chart of meta-analysis search course was shown in PRISMA 2009 Flow Diagram (S1 File). Based on the search strategy, seven literatures were satisfactory [16–22]. Table 1 shows the detailed information of all literatures.

**Table 1:** General information of eligible studies enrolled in the meta-analysis.

Study	Ethnics (Country)	Genotyping method	Control Origin	Sample capacity	Matching standard	HWE conformity	NOS
Teixeira *etal* (19)	Brazilian(Brazil)	PCR-RFLP	HB	112/180	sex, ethnicity	Yes	8
Kasyan *etal* (22)	Caucasian(Russia)	Sanger squence	HB	53/21	Age, sex, ethnicity	Yes	7
Sabrina *etal* (21)	Caucasian(Netherlands)	PCR-RFLP	PB	272/82	Age, sex, ethnicity	Yes	7
Martins *etal* (18)	Brazilian(Brazil)	PCR-RFLP	HB	107/206	Age, sex, ethnicity	Yes	8
Kluivers *etal* (16)	Caucasian(Netherlands)	PCR-RFLP	PB	204/102	Age, sex, ethnicity	Yes	8
Jeon *etal* (17)	Asian (Korea)	PCR-RFLP	PB	36/36	Age, sex, ethnicity	Yes	6
Chen *etal* (20)	Asian (Taiwan)	PCR-RFLP	HB	84/147	sex, ethnicity	Yes	7

PB: Population-based; HB: Hospital-based; HWE: Hardy-Weinberg equilibrium; RFLP: Restricted fragment length ploymorphism; NOS: Newcastle-Ottawa Scale.

### Allele and genotype-wide meta-analysis

**Fig 1:**
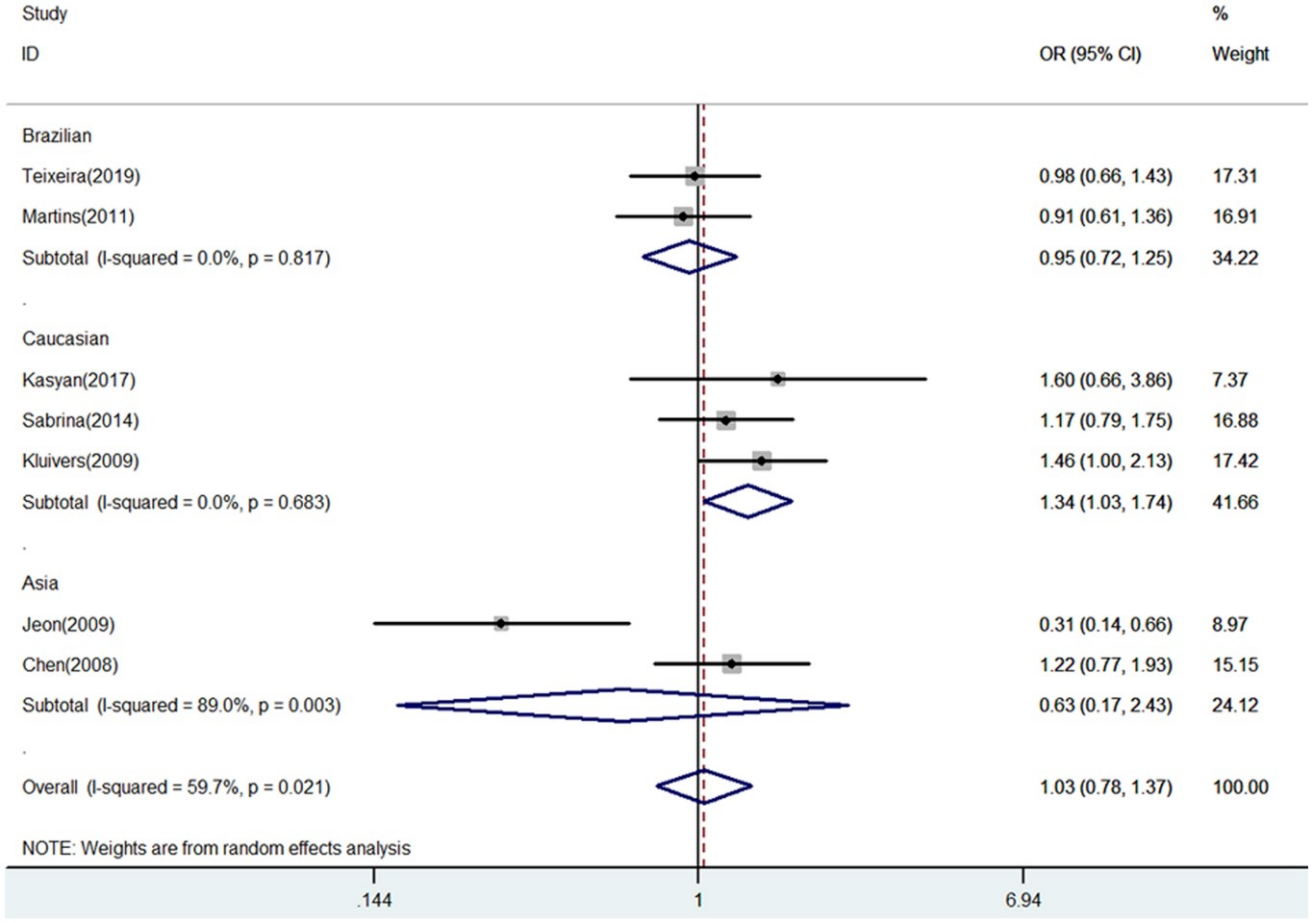
Forest plot of COL3A1 rs1800255 polymorphism on POP risk (allele contrast A vs. G). POP, pelvic organ prolapse; OR, odds ratio; CI, confidence interval.

**Fig 2:**
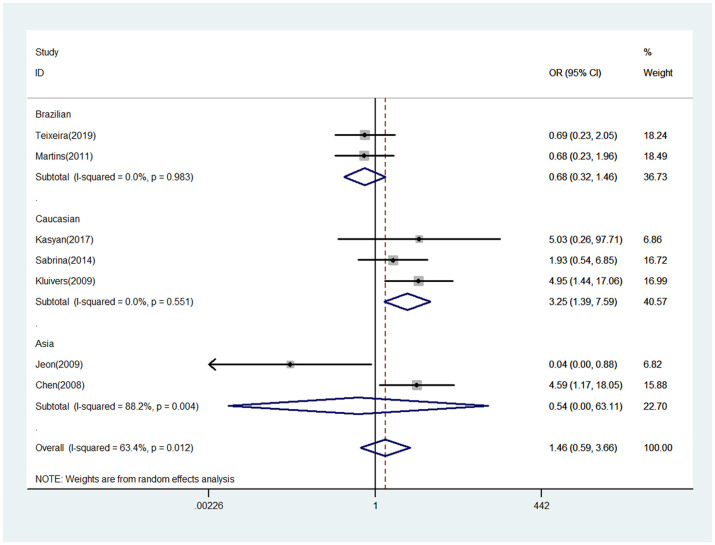
Forest plot of COL3A1 rs1800255 polymorphism on POP risk (homozygote comparison: AA vs. GG). POP, pelvic organ prolapse; OR, odds ratio; CI, confidence interval.

**Fig 3:**
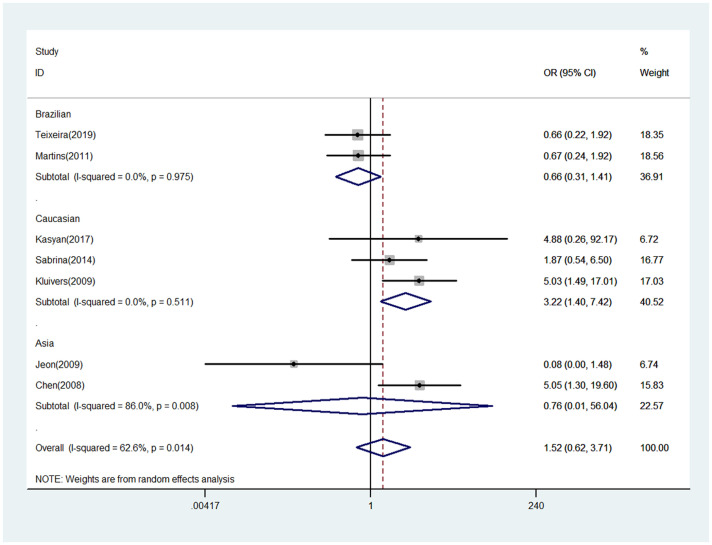
Forest plot of COL3A1 rs1800255 polymorphism on POP risk (recessive genetic model: AA vs. GG/GA). POP, pelvic organ prolapse; OR, odds ratio; CI, confidence interval.

The meta-analysis results between COL3A1 rs1800255 polymorphism and POP susceptibility are shown in Table 2. Generally, positive finding between pelvic organ prolapse and COL3A1 rs1800255 polymorphism was only found in Caucasian population by allele contrast (A vs. G: OR = 1.34, 95%CI = 1.03–1.74, P = 0.030, Fig 1), homozygote comparison (AA vs. GG: OR = 3.25, 95%CI = 1.39–7.59, P = 0.006, Fig 2), and recessive genetic model (AA vs. GG/GA: OR = 3.22, 95%CI = 1.40–7.42, P = 0.006, Fig 3).

**Table 2:** Meta-analysis of the COL3A1 rs1800255 polymorphism and POP risk.

Comparison	Population	N	Test of association			Mode	Test of heterogeneity		
			OR	95%CI	P		χ2	P	I^2^
A versus. G	Overall	7	1.03	0.78–1.37	0.827	Random	14.89	0.021	59.7
	Brazilian	2	0.95	0.72–1.25	0.689	Fixed	0.05	0.817	0
	Caucasian	3	**1.34**	**1.03–1.74**	**0.030**	Fixed	0.76	0.995	0
	Asian	2	0.63	0.17–2.43	0.506	Random	9.10	0.004	89.0
AA versus. GG	Overall	7	1.46	0.59–3.66	0.414	Random	16.40	0.012	63.4
	Brazilian	2	0.68	0.32–1.46	0.324	Fixed	0	0.983	0
	Caucasian	3	**3.25**	**1.39–7.59**	**0.006**	Fixed	1.19	0.551	0
	Asian	2	0.54	0–63.11	0.800	Random	8.48	0.004	88.2
AA versus. GA/GG	Overall	7	1.52	0.62–3.71	0.355	Random	16.04	0.171	62.6
	Brazilian	2	0.66	0.31–1.41	0.285	Fixed	0	0.788	0
	Caucasian	3	**3.22**	**1.40–7.42**	**0.006**	Fixed	1.74	0.956	0
	Asian	2	0.76	0.01–56.04	0.901	Random	7.14	0.171	86.0
AA/GA versus. GG	Overall	7	0.98	0.75–1.29	0.901	Random	9.04	0.014	33.6
	Brazilian	2	1.00	0.72–1.41	0.979	Fixed	0.07	0.975	0
	Caucasian	3	1.20	0.86–1.66	0.283	Fixed	0.09	0.511	0
	Asian	2	0.52	0.14–1.93	0.329	Random	5.46	0.008	81.7

POP, pelvic organ prolapse; OR, odds ratio; CI, confidence interval.

### Evaluation of between-study heterogeneity and sensitivity

**Fig 4:**
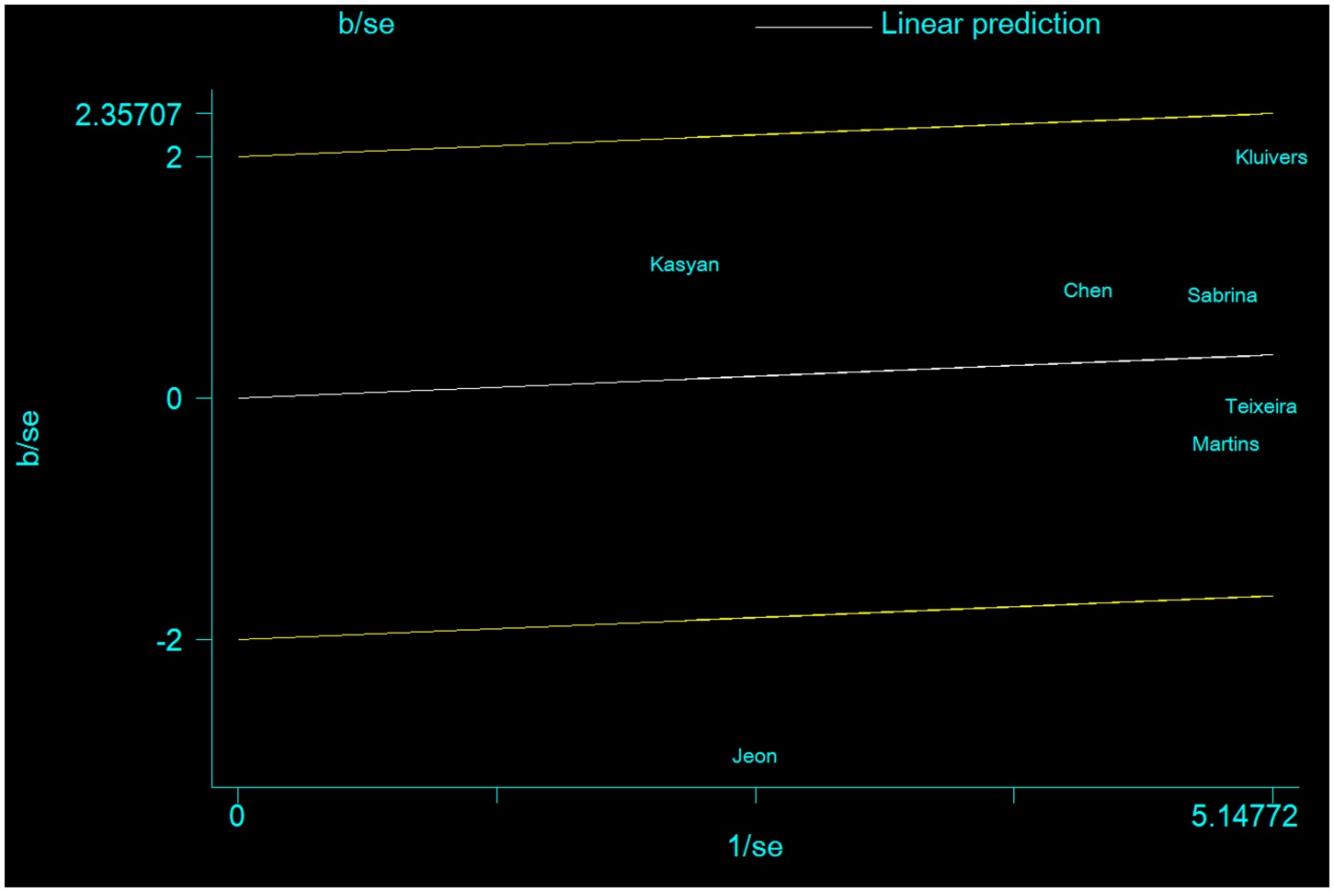
Galbraith plot of COL3A1 rs1800255 polymorphism and POP risk by allele contrast: A vs. G.

Low-Moderate heterogeneity was found under all the allele contrast (χ^2^ = 14.89, P = 0.021, I^2^ = 59.7, Table 2), homozygote comparison (χ^2^ = 16.4, P = 0.012, I^2^ = 63.4, Table 2), recessive genetic model (χ^2^ = 16.04, P = 0.171, I^2^ = 62.6, Table 2), and dominate genetic model (χ^2^ = 9.04, P = 0.014, I^2^ = 33.6, Table 2). To detect the possible source of heterogeneity, we conduct meta-regression and subgroup analysis. Meta-regression revealed that ethnicity was the main source of heterogeneity which contributed substantial heterogeneity to the final results. Then we conduct subgroup analyses stratified by ethnicity. Subsequently, the heterogeneity obviously reduced because there was no heterogeneity under all genetic models in Brazilian population and Caucasian population (all the I^2^ = 0, 0, 0, 0). However, there was still significant heterogeneity in Asian population under all genetic models (I^2^ = 89.0%, 88.2%, 86.0%, 89.0%). In order to further detect the source of heterogeneity, we conduct Galbraith plots to find out the outliers which might influence the heterogeneity. Consequently, we found that all the studies were within reasonable limits except Jeon et al (Fig 4). At last, we considered that the study Jeon et al might the reason which might contribute to the heterogeneity because its sample size was relatively too small. The small sample size might bring some bias to the final results.

### Sensitivity analysis and publication bias

**Fig 5:**
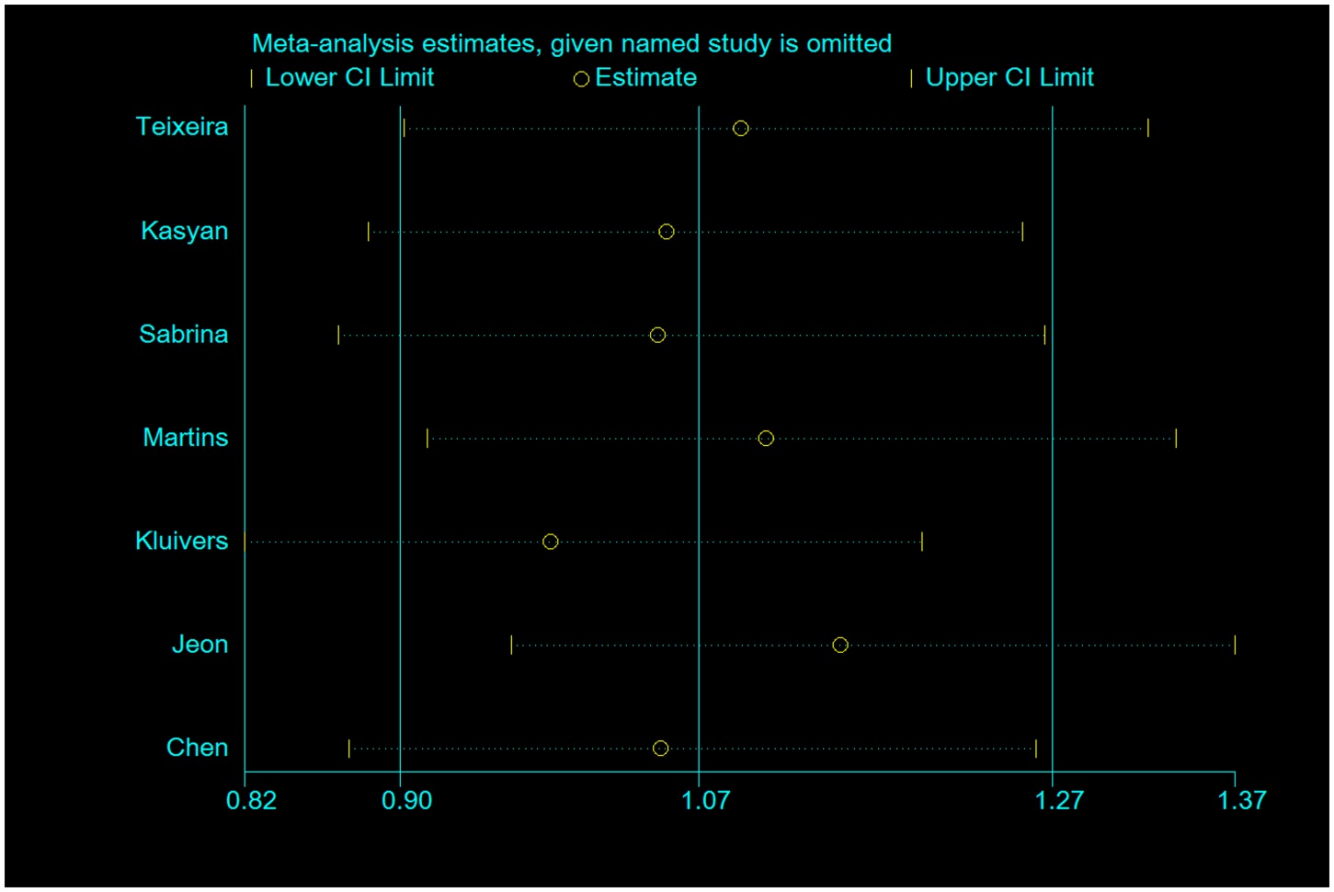
Sensitive analysis of COL3A1 rs1800255 polymorphism on POP risk (recessive genetic model: AA vs. GG/GA). POP, pelvic organ prolapse; OR, odds ratio; CI, confidence interval.

**Fig 6:**
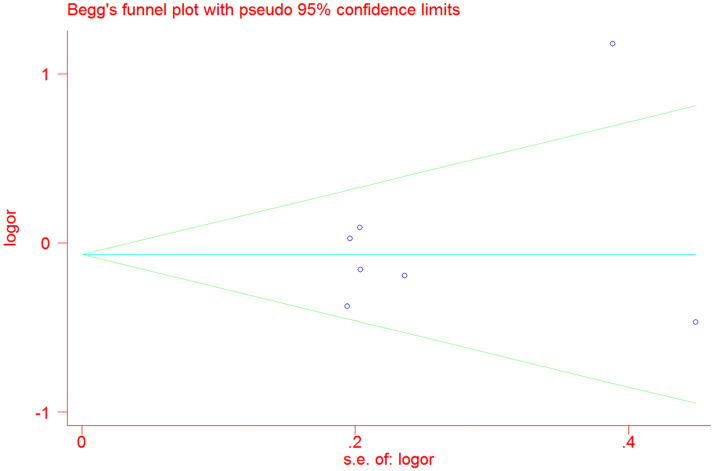
Funnel plot on publication bias for the associations between COL3A1 rs1800255 polymorphism and POP risk by allele contrast: A vs. G.

To verify the reliability and stability of meta-analysis results, sensitive analysis was applied to detect the influential studies which might contribute obvious bias to final results. The final results were not altered by removing any single literature, suggesting that the results of our meta-analysis were stable and reliable (Fig 5). We did not find obvious asymmetrical by funnel plot (P = 0.506) (Fig 6). And we also did not find any evident publication bias by Egger’s test in recessive genetic model (P = 0.820).

## Discussion

Population ageing is now a universal trend, affecting rich and poor countries in every region of the world. POP, as a common disease of elderly women, is becoming more and more prevalent. The incidence of POP is increasing in the United States. It was reported that about 40% of postmenopausal women suffered from POP with different degrees in the United States [3, 29]. The individual pelvic organ moving down from its original place not only brings about physical discomfort to patients but also brings so much inconvenience to the patient’s work and life. The patient’s work and life is affected seriously, and the quality of life decreased obviously. However, as POP has no obvious clinical symptoms, so it is easy to be ignored, leading to delay the disease treatment and bring more challenges to the work of medical workers. A random survey of Swedish women showed that only 8.3% of Swedish women had noticeable clinical symptoms of POP [30]. Some clinical symptoms seriously affect the work and life of patients, so they need surgical treatment. With the development and progress of medicine, although the surgical operation of pelvic organ prolapse is always performed by laparoscopy or vaginal operation and the trauma to patients is less than that of open surgery. However, the majority of POP patients are elderly women and the trauma has an impact on the operation tolerance and postoperative recovery ability. According to the survey, approximately 15%-18% American women needed to conduct hysterectomy because of POP, and the majority of these patients are postmenopausal women [31, 32]. Around 29,000 cases of POP in the United Kingdom required surgery each year, at a cost of 6 Million francs [33]. All of the above evidence suggests that exploring the etiology of POP contributes greatly to its diagnosis and treatment. It is well-established that the etiology of POP is awfully complicated and the role of etiology remains to be elucidated. It is well-accepted that age is a non-negligible risk factor for POP. With age, the organs of our bodies decline and degeneration with different rates. It was reported that the incidence of POP was 9.7% in women whose age is between 20~39 years old [34]. Nevertheless, the incidence considerably increased to 26.5%, 36.8% and 47.9% in women whose age is between (20~39, 40~49, 60~79) years old [34]. The results of Italian scholar indicated that with each ten-year increase in age, the POP incidence rose by about 40% [35]. It is ubiquitous that the increase by weight contributes harmful influence on health of females and it also contributes to POP pathogenesis. The studies of Bradley and Kudish suggested that obesity was risky for POP and increase of BMI bring higher morbidity [36, 37]. Another important risk factor is vaginal delivery. The literature of Luthander demonstrated that pelvic floor dysfunction is a prevalent phenomenon for women who go through vaginal delivery [38]. Some scholars from the United Arab Emirates have even suggested that POP is associated with the degree of education, income and job category [35].

If we can find the reason of pelvic floor tissue weakening, it is possible to treat the disease fundamentally. The pelvic floor tissue that supports the pelvic organs is made up of bones, muscles, fascia, and ligaments. Damage to any one of these branches can lead to POP. It is these connective tissues that play a major role in keeping all the pelvic organs in their normal anatomical positions. The elastic and retraction forces of these tissues are mainly dependent on the elastic fibers of collagen and are mainly found in the pelvic fascia. In normal adult tissues, the remodeling due to the deposition of elastic fibers is minimal, but the reproductive system has different elastic fibers, and the remodeling after childbirth is very obvious due to the overstretching of elastic fibers during pregnancy and childbirth [39]. Thus, changes in collagen content and structure will eventually lead to POP.

Apart from these non-genetic risk factors, genetic factors play a vital role in pathogenesis of POP. Compared with African-American women, the POP morbidity of Latinos and whites is 4~5 times greater. Moreover, the white race suffers from a higher morbidity than the black race and Spanish characters. These results indicate that genetic factors are crucial for POP pathogenesis. It should be noted that Cartwright et al published a literature in 2014 that also investigates the association between COL3A1 rs1800255 polymorphism and POP risk [12]. Regrettably, only two studies were included in their meta-analysis. They concluded that COL3A1 rs1800255 polymorphism was not associated with POP risk. Their result was inconsistent with ours. The results of our meta-analysis showed COL3A1 rs1800255 polymorphism contributes no risk to POP of Brazilian and Asian population but contributes increased risk to POP of Caucasian population. There is no doubt that our study reduces the bias and the result is more reliable.

It is important to emphasize the importance of collagen. Collagen is the main component of extracellular matrix and plays an important role in pelvic floor supporting tissue. No matter what mechanism causes pelvic organ prolapse, the ultimate reason will be attributed to the decrease of collagen, which causes the occurrence of pelvic organ prolapse. So far, type III collagen is the most widely investigated collagen type in pelvic connective structures, which plays an important role in the formation of vaginal wall. Type III collagen, encoded by COL3A1 gene, consists of three identical α1 (III) chains. The genetic polymorphisms of COL3A1 gene lead to amino acid change of the α1 (III) chain. This COL3A1 rs1800255 polymorphism is a G/A variation and located in position 2209 (exon 30). The above exchange leads to change of protein-level expression (alanine/threonine). And threonine has a larger and more hydrophilic side chain than alanine, leading to disturb the triple helical conformation of type III collagen. In recent years, N4-acetylcytidine (ac4C) has become a research hotspot and has been paid wide attention. Recent studies have shown extensive ac4C modifications in human and yeast mRNAs [40]. And ac4C helps to correctly read codons during translation and improves translation efficiency and the stability of mRNA [40]. Also, ac4C is associated with the development, progression, and prognosis of a variety of human diseases including metabolic diseases, inflammatory diseases, autoimmune diseases and cancer [40]. It can be studied in the future whether ac4C could influence on COL3A1 mRNA expression.

This is, to our knowledge, the first meta-analysis of genetic studies on the association between POP risk and COL3A1 rs1800255 polymorphism. We found that COL3A1 rs1800255 polymorphism contributes no risk to POP of Brazilian and Asian population but contributes increased risk to POP of Caucasian population. To be specific, the A allele and AA genotype were risk factors and they contribute a increased risk to POP risk in Caucasian population. Eliminating the source of bias is of vital importance for gene polymorphism association meta-analysis. Hence, we have attempted to conduct all the three patterns in the present meta-analysis. Firstly, allele contrast was used to find out the high risk or low risk allele. Secondly, homozygote comparison was used to find out the high risk or low risk genotype. The last pattern is comparing homozygote genotype versus allele carriers. In the present study, the low-moderate heterogeneity between studies occurred in the overall population. Common reasons for heterogeneity consist of differences in the investigated populations or in genotyping methods or in sample size or it may be derived from other risk factors. By performing meta-regression, subgroup analysis and Galbraith plot analysis, we found that ethnicity and sample size might contribute substantial heterogeneity to final results. In the present study, we successfully find out the source of heterogeneity. And the results of sensitive analysis and publication bias demonstrated that the results of our meta-analysis were stable and reliable.

Some limitations should be pointed out. Firstly, variable confounding factors including aging, obesity, vaginal delivery, hypoestrogenism and preoperative hysterectomy are not fully considered due to limited dataset [41, 42], therefore, further investigations on this gene taking these factors into consideration for these possible confounding factors should be conducted for subgroup analysis the future [43, 44]. Secondly, although we know that only one SNP within COL3A1 is not enough to elucidate the role of this gene on the susceptibility to POP, however, the number of literatures investigating the association between other COL3A1 SNP sites and POP susceptibility is considerably rare. For example, only one literature investigates the association between COL3A1 rs1801184 polymorphism and POP susceptibility. Thirdly, the association between COL3A1 rs1800255 polymorphism and POP risk has not been investigated in GWAS. So that well-designed GWAS should be included for further trans-ethnic and trans-trait meta-analysis [45]. If multiple independent SNPs within other genomic regions along with this COL3A1 rs1801184 polymorphism and environmental factors can be well investigated in large GWAS data, we may construct a machine-learning prediction model for early diagnosis of the disease in the framework of precision medicine [46, 47]. Lastly, we did not conduct functional investigation to disclose the causal effect of this potential risk genetic variant on the development of POP, but a very useful strategy named mendelian randomization, to integrating the COL3A1 genotype data with eQTL from GTEX or pQTLs, may explore the statistical causality of the genetic variant rs1801184 in the development of POP [48, 49]. Due to the lack of the gene expression in specific tissues, it is hard to test if the COL3A1 rs180025 polymorphism or other SNPs in this gene are causally triggering the development of POP through mediating the expression of this gene in specific tissues.

In summary, the present meta-analysis suggests that the COL3A1 is a potential risk gene for POP. Caucasian individuals with COL3A1 rs1800255 A allele and AA genotype have a higher risk of POP. The COL3A1 rs1800255 polymorphism may be a risk factor for POP in Caucasian population. Further studies investigating other confirmed genetic factors and possible gene-gene and gene-environmental interactions for COL3A1 rs1800255 polymorphism in a large GWAS data should be warranted.

## Supporting information

S1 Table PRISMA 2009 checklist. (DOC)

S1 File PRISMA 2009 flow diagram. (DOC)

## References

[1] Fang G, Hong L, Liu C, Yang Q, Zhang Q, et al. Oxidative status of cardinal ligament in pelvic organ prolapse. Experimental and therapeutic medicine.2018; 16: 3293–3302. 10.3892/etm.2018.6633 30250520 PMC6143997

[2] Han L, Wang L, Wang Q, Li H, Zang H. Association between pelvic organ prolapse and stress urinary incontinence with collagen. Experimental and therapeutic medicine.2014; 7: 1337–1341. 10.3892/etm.2014.1563 24940435 PMC3991483

[3] Hendrix SL, Clark A, Nygaard I, Aragaki A, Barnabei V, et al. Pelvic organ prolapse in the Women’s Health Initiative: gravity and gravidity. American journal of obstetrics and gynecology.2002; 186: 1160–1166. 10.1067/mob.2002.123819 12066091

[4] Wu JM, Vaughan CP, Goode PS, Redden DT, Burgio KL, et al. Prevalence and trends of symptomatic pelvic floor disorders in U.S. women. Obstetrics and gynecology.2014; 123: 141–148. 10.1097/AOG.0000000000000057 24463674 PMC3970401

[5] Chen J, Zhao X, Cui L, He G, Wang X, et al. Genetic regulatory subnetworks and key regulating genes in rat hippocampus perturbed by prenatal malnutrition: implications for major brain disorders. Aging.2020; 12: 8434–8458. 10.18632/aging.103150 32392183 PMC7244046

[6] Islam RM, Oldroyd J, Rana J, Romero L, Karim MN. Prevalence of symptomatic pelvic floor disorders in community-dwelling women in low and middle-income countries: a systematic review and meta-analysis. International urogynecology journal.2019; 30: 2001–2011. 10.1007/s00192-019-03992-z 31165221

[7] Li H, Wang X, Lu X, Zhu H, Li S, et al. Co-expression network analysis identified hub genes critical to triglyceride and free fatty acid metabolism as key regulators of age-related vascular dysfunction in mice. Aging.2019; 11: 7620–7638. 10.18632/aging.102275 31514170 PMC6781998

[8] Wang X, Jiao X, Tian Y, Zhang J, Zhang Y, et al. Associations between maternal vitamin D status during three trimesters and cord blood 25(OH)D concentrations in newborns: a prospective Shanghai birth cohort study. European journal of nutrition.2021. 10.1007/s00394-021-02528-w 33661376

[9] Zheng S, Zhao T, Yuan S, Yang L, Ding J, et al. Immunodeficiency Promotes Adaptive Alterations of Host Gut Microbiome: An Observational Metagenomic Study in Mice. Frontiers in microbiology.2019; 10: 2415. 10.3389/fmicb.2019.02415 31781050 PMC6853035

[10] Lince SL, van Kempen LC, Vierhout ME, Kluivers KB. A systematic review of clinical studies on hereditary factors in pelvic organ prolapse. International urogynecology journal.2012; 23: 1327–1336. 10.1007/s00192-012-1704-4 22422218 PMC3448053

[11] Ward RM, Velez Edwards DR, Edwards T, Giri A, Jerome RN, et al. Genetic epidemiology of pelvic organ prolapse: a systematic review. American journal of obstetrics and gynecology.2014; 211: 326–335. 10.1016/j.ajog.2014.04.006 24721264 PMC4213176

[12] Cartwright R, Kirby AC, Tikkinen KA, Mangera A, Thiagamoorthy G, et al. Systematic review and metaanalysis of genetic association studies of urinary symptoms and prolapse in women. American journal of obstetrics and gynecology.2015; 212: 199.e191–124. 10.1016/j.ajog.2014.08.005 25111588 PMC4342521

[13] Lim VF, Khoo JK, Wong V, Moore KH. Recent studies of genetic dysfunction in pelvic organ prolapse: the role of collagen defects. The Australian & New Zealand journal of obstetrics & gynaecology.2014; 54: 198–205. 10.1111/ajo.12169 24575973

[14] Liu SH, Yang RS, al-Shaikh R, Lane JM. Collagen in tendon, ligament, and bone healing. A current review. Clinical orthopaedics and related research.1995: 265–278. 7671527

[15] Ricard-Blum S. The collagen family. Cold Spring Harbor perspectives in biology.2011; 3: a004978. 10.1101/cshperspect.a004978 21421911 PMC3003457

[16] Kluivers KB, Dijkstra JR, Hendriks JC, Lince SL, Vierhout ME, et al. COL3A1 2209G>A is a predictor of pelvic organ prolapse. International urogynecology journal and pelvic floor dysfunction.2009; 20: 1113–1118. 10.1007/s00192-009-0913-y 19444361

[17] Jeon MJ, Chung SM, Choi JR, Jung HJ, Kim SK, et al. The relationship between COL3A1 exon 31 polymorphism and pelvic organ prolapse. The Journal of urology.2009; 181: 1213–1216. 10.1016/j.juro.2008.11.027 19152942

[18] Martins Kde F, de Jarmy-DiBella ZI, da Fonseca AM, Castro RA, da Silva ID, et al. Evaluation of demographic, clinical characteristics, and genetic polymorphism as risk factors for pelvic organ prolapse in Brazilian women. Neurourology and urodynamics.2011; 30: 1325–1328. 10.1002/nau.21066 21608022

[19] Teixeira FH, Fernandes CE, do Souto RP, de Oliveira E. Polymorphism rs1800255 from COL3A1 gene and the risk for pelvic organ prolapse. International urogynecology journal.2020; 31: 73–78. 10.1007/s00192-019-03965-2 31041498

[20] Chen HY, Chung YW, Lin WY, Wang JC, Tsai FJ, et al. Collagen type 3 alpha 1 polymorphism and risk of pelvic organ prolapse. International journal of gynaecology and obstetrics: the official organ of the International Federation of Gynaecology and Obstetrics.2008; 103: 55–58. 10.1016/j.ijgo.2008.05.031 18722615

[21] Lince SL, van Kempen LC, Dijkstra JR, IntHout J, Vierhout ME, et al. Collagen type III alpha 1 polymorphism (rs1800255, COL3A1 2209 G>A) assessed with high-resolution melting analysis is not associated with pelvic organ prolapse in the Dutch population. International urogynecology journal.2014; 25: 1237–1242. 10.1007/s00192-014-2385-y 24760181

[22] Kasyan GR, Vishnevskii DA, Akulenko LV, Kozlova YO, Sharova EI, et al. [Association of polymorphism of 1800255 COL3A1 gene with pelvic organ prolapse and urinary incontinence in women: preliminary data]. Urologiia.2017: 30–33. 29376591

[23] Stang A. Critical evaluation of the Newcastle-Ottawa scale for the assessment of the quality of nonrandomized studies in meta-analyses. European journal of epidemiology.2010; 25: 603–605. 10.1007/s10654-010-9491-z 20652370

[24] Higgins JP, Thompson SG, Deeks JJ, Altman DG. Measuring inconsistency in meta-analyses. Bmj.2003; 327: 557–560. 10.1136/bmj.327.7414.557 12958120 PMC192859

[25] DerSimonian R, Laird N. Meta-analysis in clinical trials. Controlled clinical trials.1986; 7: 177–188. 10.1016/0197-2456(86)90046-2 3802833

[26] Mantel N, Haenszel W. Statistical aspects of the analysis of data from retrospective studies of disease. Journal of the National Cancer Institute.1959; 22: 719–748. 13655060

[27] Begg CB, Mazumdar M. Operating characteristics of a rank correlation test for publication bias. Biometrics.1994; 50: 1088–1101. 7786990

[28] Moher D, Liberati A, Tetzlaff J, Altman DG, Group P. Preferred reporting items for systematic reviews and meta-analyses: the PRISMA statement. Journal of clinical epidemiology.2009; 62: 1006–1012. 10.1016/j.jclinepi.2009.06.005 19631508

[29] Handa VL, Garrett E, Hendrix S, Gold E, Robbins J. Progression and remission of pelvic organ prolapse: a longitudinal study of menopausal women. American journal of obstetrics and gynecology.2004; 190: 27–32. 10.1016/j.ajog.2003.07.017 14749630

[30] Tegerstedt G, Maehle-Schmidt M, Nyren O, Hammarstrom M. Prevalence of symptomatic pelvic organ prolapse in a Swedish population. International urogynecology journal and pelvic floor dysfunction.2005; 16: 497–503. 10.1007/s00192-005-1326-1 15986100

[31] Lepine LA, Hillis SD, Marchbanks PA, Koonin LM, Morrow B, et al. Hysterectomy surveillance—United States, 1980–1993. MMWR CDC surveillance summaries: Morbidity and mortality weekly report CDC surveillance summaries.1997; 46: 1–15. 9259214

[32] Popovic JR, Kozak LJ. National hospital discharge survey: annual summary, 1998. Vital and health statistics Series 13, Data from the National Health Survey.2000: 1–194. 11077892

[33] Elenskaia K, Thakar R, Sultan AH, Scheer I, Onwude J. Effect of childbirth on pelvic organ support and quality of life: a longitudinal cohort study. International urogynecology journal.2013; 24: 927–937. 10.1007/s00192-012-1932-7 22955252

[34] Nygaard I, Barber MD, Burgio KL, Kenton K, Meikle S, et al. Prevalence of symptomatic pelvic floor disorders in US women. Jama.2008; 300: 1311–1316. 10.1001/jama.300.11.1311 18799443 PMC2918416

[35] Krissi H, Aviram A, Eitan R, From A, Wiznitzer A, et al. Risk factors for recurrence after Le Fort colpocleisis for severe pelvic organ prolapse in elderly women. International journal of surgery.2015; 20: 75–79. 10.1016/j.ijsu.2015.06.026 26079498

[36] Bradley CS, Zimmerman MB, Qi Y, Nygaard IE. Natural history of pelvic organ prolapse in postmenopausal women. Obstetrics and gynecology.2007; 109: 848–854. 10.1097/01.AOG.0000255977.91296.5d 17400845

[37] Kinman CL, Lemieux CA, Agrawal A, Gaskins JT, Meriwether KV, et al. The relationship between age and pelvic organ prolapse bother. International urogynecology journal.2017; 28: 751–755. 10.1007/s00192-016-3175-5 27766345

[38] Luthander C, Emilsson T, Ljunggren G, Hammarstrom M. A questionnaire on pelvic floor dysfunction postpartum. International urogynecology journal.2011; 22: 105–113. 10.1007/s00192-010-1243-9 20798924 PMC3018591

[39] Downing KT, Billah M, Raparia E, Shah A, Silverstein MC, et al. The role of mode of delivery on elastic fiber architecture and vaginal vault elasticity: a rodent model study. Journal of the mechanical behavior of biomedical materials.2014; 29: 190–198. 10.1016/j.jmbbm.2013.08.025 24099948 PMC3857332

[40] Jin G, Xu M, Zou M, Duan S. The Processing, Gene Regulation, Biological Functions, and Clinical Relevance of N4-Acetylcytidine on RNA: A Systematic Review. Molecular therapy Nucleic acids.2020; 20: 13–24. 10.1016/j.omtn.2020.01.037 32171170 PMC7068197

[41] Yan X, Zhao X, Li J, He L, Xu M. Effects of early-life malnutrition on neurodevelopment and neuropsychiatric disorders and the potential mechanisms. Progress in neuro-psychopharmacology & biological psychiatry.2018; 83: 64–75. 10.1016/j.pnpbp.2017.12.016 29287829

[42] Zhou X, Li Q, Xu J, Zhang X, Zhang H, et al. The aberrantly expressed miR-193b-3p contributes to preeclampsia through regulating transforming growth factor-beta signaling. Scientific reports.2016; 6: 19910. 10.1038/srep19910 26822621 PMC4731805

[43] Xu MQ, Ye Z, Hu FB, He L. Quantitative assessment of the effect of angiotensinogen gene polymorphisms on the risk of coronary heart disease. Circulation.2007; 116: 1356–1366. 10.1161/CIRCULATIONAHA.107.728857 17846284

[44] Jiang L, Wang K, Lo K, Zhong Y, Yang A, et al. Sex-Specific Association of Circulating Ferritin Level and Risk of Type 2 Diabetes: A Dose-Response Meta-Analysis of Prospective Studies. The Journal of clinical endocrinology and metabolism.2019; 104: 4539–4551. 10.1210/jc.2019-00495 31074789

[45] Wu Y, Cao H, Baranova A, Huang H, Li S, et al. Multi-trait analysis for genome-wide association study of five psychiatric disorders. Translational psychiatry.2020; 10: 209. 10.1038/s41398-020-00902-6 32606422 PMC7326916

[46] Yu H, Pan R, Qi Y, Zheng Z, Li J, et al. LEPR hypomethylation is significantly associated with gastric cancer in males. Experimental and molecular pathology.2020; 116: 104493. 10.1016/j.yexmp.2020.104493 32659237

[47] Liu M, Li F, Yan H, Wang K, Ma Y, et al. A multi-model deep convolutional neural network for automatic hippocampus segmentation and classification in Alzheimer’s disease. NeuroImage.2020; 208: 116459. 10.1016/j.neuroimage.2019.116459 31837471

[48] Hou L, Xu M, Yu Y, Sun X, Liu X, et al. Exploring the causal pathway from ischemic stroke to atrial fibrillation: a network Mendelian randomization study. Molecular medicine.2020; 26: 7. 10.1186/s10020-019-0133-y 31941463 PMC6964084

[49] Cai L, Bao Y, Fu X, Cao H, Baranova A, et al. Causal links between major depressive disorder and insomnia: A Mendelian randomisation study. Gene.2021; 768: 145271. 10.1016/j.gene.2020.145271 33122081

