# Supplementary material for: COL3A1 rs1800255 polymorphism is associated with pelvic organ prolapse susceptibility in Caucasian individuals: Evidence from a meta-analysis
Source: PLoS One. 2021 Apr 30;16(4):e0250943. doi: 10.1371/journal.pone.0250943 (PMC8087080; doi:10.1371/journal.pone.0250943)
Supplement: S1 File — (DOC) [file pone.0250943.s002.doc]

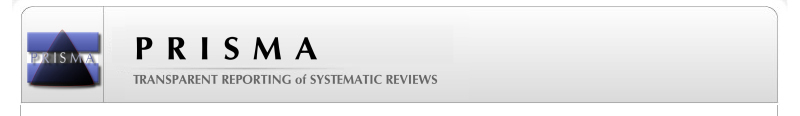
**PRISMA 2009 Flow Diagram**

**Screening**

**Included**

**Eligibility**

**Identification**

Records identified through database searching
(n = 15 )

Additional records identified through other sources
(n = 0 )

Records after duplicates removed
(n =15 )

Records screened
(n =9 )

Records excluded
(n = 6 )

Full-text articles assessed for eligibility
(n = 7 )

Full-text articles excluded, with reasons
(n = 2 )

Studies included in qualitative synthesis
(n = 7 )

Studies included in quantitative synthesis (meta-analysis)
(n = 7 )
